# Reconstruction of Full-Length circRNA Sequences Using Chimeric Alignment Information

**DOI:** 10.3390/ijms23126776

**Published:** 2022-06-17

**Authors:** Md. Tofazzal Hossain, Jingjing Zhang, Md. Selim Reza, Yin Peng, Shengzhong Feng, Yanjie Wei

**Affiliations:** 1 Center for High Performance Computing, Joint Engineering Research Center for Health Big Data Intelligent Analysis Technology, Shenzhen Institute of Advanced Technology, Chinese Academy of Sciences, Shenzhen 518055, China; tofazzal.stat@gmail.com (M.T.H.); zhangjj@siat.ac.cn (J.Z.); selim@siat.ac.cn (M.S.R.); sz.feng@siat.ac.cn (S.F.); 2 School of Computer Science and Technology, University of Chinese Academy of Sciences, Beijing 100049, China; 3 Department of Statistics, Bangabandhu Sheikh Mujibur Rahaman Science and Technology University, Gopalganj 8100, Bangladesh; 4 Department of Pathology, The Shenzhen University School of Medicine, Shenzhen 518060, China

**Keywords:** circular RNA, reconstruction of circRNA sequence, full-length sequence

## Abstract

Circular RNAs (circRNAs) are RNA molecules formed by joining a downstream 3 splice donor site and an upstream 5 splice acceptor site. Several recent studies have identified circRNAs as potential biomarker for different diseases. A number of methods are available for the identification of circRNAs. The circRNA identification methods cannot provide full-length sequences. Reconstruction of the full-length sequences is crucial for the downstream analyses of circRNA research including differential expression analysis, circRNA-miRNA interaction analysis and other functional studies of the circRNAs. However, a limited number of methods are available in the literature for the reconstruction of full-length circRNA sequences. We developed a new method, circRNA-full, for full-length circRNA sequence reconstruction utilizing chimeric alignment information from the STAR aligner. To evaluate our method, we used full-length circRNA sequences produced by isocirc and ciri-long using long-reads RNA-seq data. Our method achieved better reconstruction rate, precision, sensitivity and F1 score than the existing full-length circRNA sequence reconstruction tool ciri-full for both human and mouse data.

## 1. Introduction

Circular RNAs (circRNAs) are RNA molecules with covalently closed RNA loops, produced by joining a downstream 3′ splice donor site and an upstream 5′ splice acceptor site [1,2,3]. They are generated from both coding and noncoding linear transcripts [4]. They can comprise exonic or intronic sequences from their parent genes, and their sizes may vary from less than 100 nt to more than 4000 nt [5]. CircRNAs can originate from exons, introns, antisense, intergenic regions and 5′ or 3′ untranslated regions [6]. They are produced through a variety of distinct mechanisms depending on complementary sequences within flanking introns [7,8,9], exon skipping [9,10], and exon-containing lariat precursors [11]. Among several important properties, circRNAs have the ability to effectively sponge miRNAs [12], can interact with proteins [13] and can be translated into functional proteins [14]. CircRNAs can also interact with RNA-binding proteins (RBPs) [15], but there is minimal enrichment in RBP binding sites for circRNA sequences compared to their linear mRNA counterparts. Several studies have demonstrated that circRNA transcripts can be involved in gene regulation [16], development [17], natural immune responses [18] and diseases [19,20]. A number of studies have identified circRNAs as potential biomarkers for cancers [21,22,23], autoimmune diseases [24,25], atherosclerosis [26], disorders of the central neural diseases [27], and degenerative diseases [28].

In the literature, a number of methods are available for the identification of circRNAs including find_circ [29], CIRCexplorer [8], CIRI [30], circRNA_finder [31], and DCC [32], among others. All of these methods identify circRNAs with their start and end position in the chromosome but cannot provide full-length circRNA sequences. The full-length sequence of the circRNA is crucial for downstream analyses in circRNA research, including differential expression analysis, circRNA-miRNA interaction analysis and other functional studies of the circRNAs. A limited number of methods, including ciri-full [33], FUCHS [34], and FcircSEC [35] are available for full-length circRNA sequence reconstruction. To assemble full-length circRNA sequences, ciri-full uses both back-splice junction (BSJ) sites and reverse overlap (RO) features. The output of CIRI and RNA-seq data are needed for ciri-full to assemble the full-length sequence. FUCHS is designed to fully characterize a putative circRNA sequence using RNA-seq information from long reads (>150 bp). It utilizes the internal components of the BSJ and produces the circRNA sequences using the mapping results of BSJ reads. On the other hand, FcircSEC is developed for the full-length circRNA sequence reconstruction and classification using the annotation information of the reference genome, and fully depends on the annotation information. Our method circRNAfull utilizes chimeric alignment information of the spanning reads of the circRNA. The key drawback of ciri-full is that it cannot be used if the sequencing read lengths for all reads in the RNA-seq data are not equal. Moreover, the annotation file in gff format is not accepted by ciri-full. FUCHS is tested with the output of DCC only and is not suitable for short reads, and it does not provide the full-length sequence directly. FcircSEC is based on annotation information of the reference genome and does not use sequencing reads. Mostly, it combines all possible exons within the circRNA boundary for exonic circRNAs. It depends on an assumption that circular and linear transcripts share the same composition. However, this assumption is unsupported and may lead to misunderstanding in downstream analyses [33]. To address these limitations, we developed a method, circRNA-full, for reconstructing full-length circRNA sequences utilizing the chimeric alignments of the STAR aligner.

In this paper, we present a new method, circRNA-full, using chimeric alignments of the STAR aligner together with the output of CIRCexplorer. Comparing the performance of circRNA-full with ciri-full in terms of precision, sensitivity and F1 score, we found that circRNA-full performed better than ciri-full for both human and mouse data.

## 2. Results

### 2.1. Identification of circRNAs

**Figure 1:**
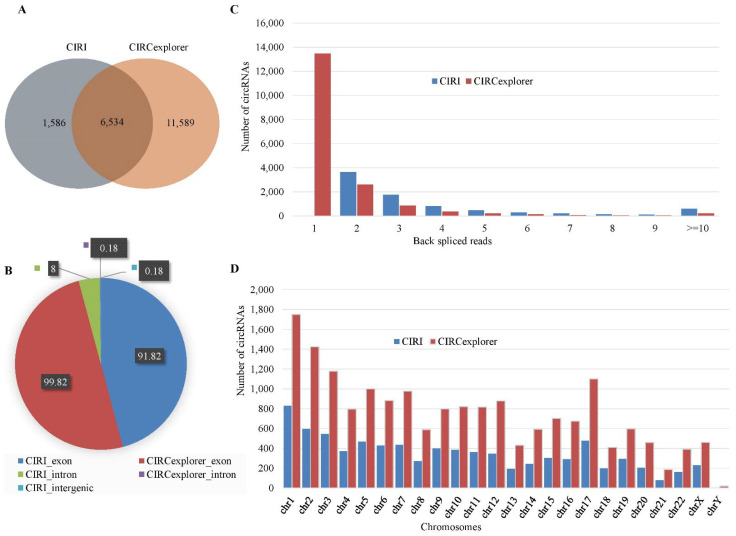
Expression profile of circRNAs in human data. (**A**) Venn-diagram of the circRNAs identified by CIRI and CIRCexplorer. (**B**) Distribution of the genomic origin of the circRNAs (%). (**C**) Distribution of the number of back-spliced reads spanning the circRNAs. (**D**) Chromosome distribution of the identified circRNAs.

We identified circRNAs using two popular tools: CIRI (version 2) and CIRCexplorer (version 2). For human data, a total of 8120 circRNAs were identified by CIRI, whereas a total of 18,123 circRNAs were identified by CIRCexplorer. The number of common circRNAs identified both algorithms was 6534 (Figure 1A). A total of 13,175 circRNAs were identified by a single algorithm. Among the circRNAs identified by CIRI, 91.82% (7456/8120) were exonic, 8% (650/8120) were intronic and the rest were intergenic (Figure 1B). The average number of back-splice reads was five. The distribution of back-splice reads showed that 77% of the circRNAs were supported by less than or equal to four back-splice reads (Figure 1C). The chromosome distribution was heterogeneous and showed that the maximum number (831) of circRNAs were originated from chromosome 1 (Figure 1D). For the circRNAs identified by CIRCexplorer, there were 99.82% (18,091/18,123) exonic circRNAs and the rest were intronic. The average number of back-splice reads was two. The majority of the circRNAs (88.78%) were supported by less than or equal to two back-splice reads. The chromosome distribution was heterogeneous and the maximum number (1749) of circRNAs were originated from chromosome 1. Figure 1 showed the expression profile of circRNAs for human data.

**Figure 2:**
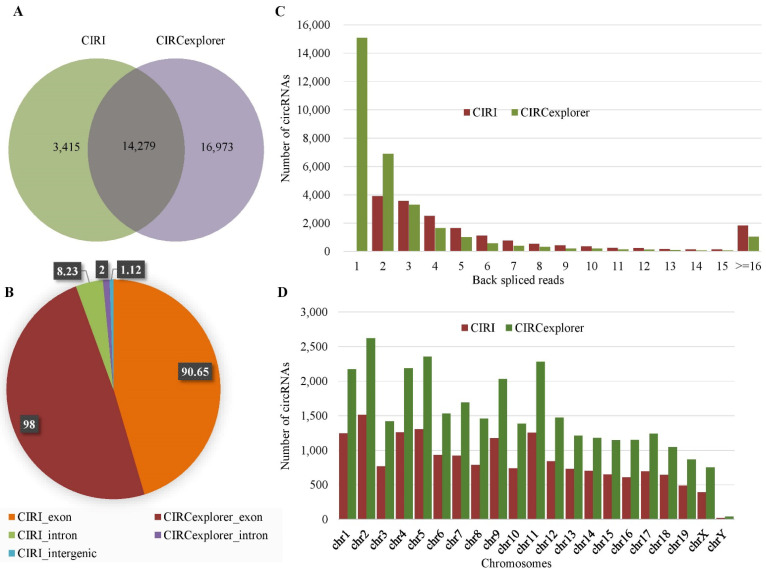
Expression profile of circRNAs in mouse data. (**A**) Venn-diagram of the circRNAs identified by CIRI and CIRCexplorer. (**B**) Distribution of the genomic origin of the circRNAs (%). (**C**) Distribution of the number of back-spliced reads spanning the circRNAs. (**D**) Chromosome distribution of the identified circRNAs.

For mouse data, 17,694 circRNAs were identified by CIRI, whereas 31,252 circRNAs were identified by CIRCexplorer. We found a total of 14,279 circRNAs were identified by both the tools, whereas 20,388 circRNAs were identified by a single method (Figure 2A). There were 90.65% (16,040/17,694) exonic, 8.23% (1456/17,694) intronic, and the rest were intergenic circRNAs among the total circRNAs identified by CIRI (Figure 2B). The average number of back-splice reads spanning the circRNAs was ten. Around 77% of the circRNAs were supported by less than or equal to seven back-splice reads. From the chromosome distribution, it was found that the distribution was heterogeneous and the maximum number of circRNAs (1513) were generated from chromosome 2 (chr2) (Figure 2D). Among the circRNAs identified by CIRCexplorer, 98% (30,756/31,252) were exonic and the rest were intronic, and no intergenic circRNAs were found. The average number of back-splice reads was four and about 81% circRNAs were spanned by less than or equal to three back-splice reads. The distribution of circRNAs in different chromosomes was heterogeneous and most circRNAs were in chromosome 2 (chr2). Figure 2 showed the expression profile of circRNAs for mouse data.

We found that more circRNAs were predicted by CIRCexplorer than CIRI in human and mouse data. No circRNAs identified by CIRI were spanned by less than two back-spliced reads, whereas 74% and 48% of the circRNAs identified by CIRCexplorer were spanned by only one back-splice read in human and mouse data respectively. Moreover, the circRNAs produced by CIRI were supported by larger average number of back-splice reads than that of the circRNAs produced by CIRCexplorer in both datasets. More circRNAs were identified in mouse than human, and the average number of back-splice reads per circRNA was larger in mouse than human.

### 2.2. Reconstruction of Full-Length Sequence

We reconstructed full-length sequences of the circRNAs using the ciri-full and our developed method circRNA-full. We used two tools, CIRI and CIRCexplorer, for the prediction of circRNAs. For reconstructing the full-length sequence, we used those circRNAs which were identified by both CIRI and CIRCexplorer. The reason was to use the same set of circRNAs for the performance comparison of ciri-full and circRNA-full.

**Figure 3:**
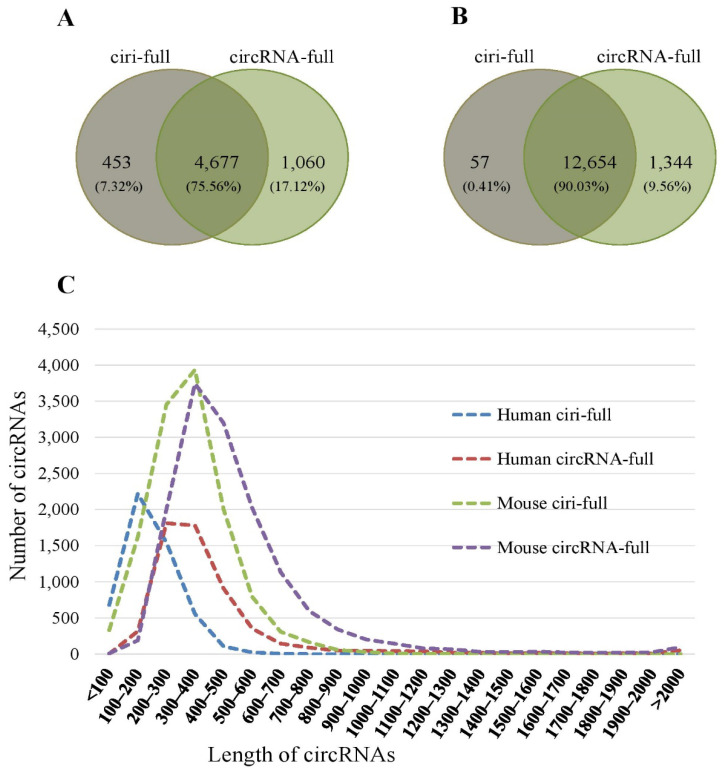
Sequence reconstruction results for circRNA-full and ciri-full. (**A**) Venn-diagram of the number of reconstructed circRNAs for human data. (**B**) Venn-diagram of the number of reconstructed circRNAs for the mouse data. (**C**) Length distribution of reconstructed circRNAs.

For human data, a total of 5130 full-length circRNAs were assembled by ciri-full, whereas 5737 full-length circRNAs were assembled by our method, circRNA-full. Among these full-length circRNAs, 75.56% of the circRNAs were assembled by both ciri-full and circRNA-full, whereas 7.32% of the circRNAs were assembled by ciri-full only, and 17.12% of the circRNAs were assembled by circRNA-full only (Figure 3A). For mouse data, 12,711 full-length circRNAs were assembled by ciri-full, whereas 13,998 full-length circRNAs were assembled by circRNA-full. Moreover, 90.03% of the circRNAs were assembled by both ciri-full and circRNA-full (Figure 3B). Of the circRNAs, 0.41% and 9.56% were assembled by the single assembly methods ciri-full and circRNA-full, respectively (Figure 3B). More full-length circRNAs were assembled in mouse than human.

From Table 1, it can be observed that the assembly rate of circRNA-full was more than that of the ciri-full. For sample SRR10612068, 81.15% of the circRNAs were assembled by ciri-full, whereas 90.81% of the circRNAs were assembled by circRNA-full. For mouse data, in sample CRR194214, 94.95% of the circRNAs were reconstructed by ciri-full, whereas 99.58% of the circRNAs were reconstructed by circRNA-full. Between human and mouse data, the percentage of assembled sequences was more in the mouse data. From Figure 3C, we can observe that the length of most of the circRNAs were less than 1000 nt. The length of the circRNAs reconstructed by circRNA-full was longer than that of the circRNAs produced by ciri-full in both human and mouse data. Again, the length of the circRNAs in mouse data was longer than that of the circRNAs in human data.

For human data, it was found that the length of circRNAs produced by circRNA-full (median 334) was greater than that of the circRNAs produced by ciri-full (median 182), and the number was statistically significant as determined by the Mann-Whitney U-test ($p<2.20\times 10^{-16}$). We also observed that 75% of the circRNAs had a length of 432 nt for circRNA-full and of 250 nt for ciri-full. For mouse data, the length of circRNAs produced by circRNA-full (median 428) was greater than that of the circRNAs produced by ciri-full (median 320) with $p<2.20\times 10^{-16}$ determined by the Mann-Whitney U-test. Again 75% of the circRNAs had a length of 559 nt for circRNA-full and of 406 nt for ciri-full. For combined data (human and mouse) the length of circRNAs generated by circRNA-full (median 400) was greater than that of the circRNAs generated by ciri-full (median 283), which was significant as determined by Mann-Whitney U-test ($p<2.20\times 10^{-16}$). Seventy-five percent of the circRNAs had lengths of less than 529 nt for circRNA-full and less than 372 nt for ciri-full.

### 2.3. Performance Comparison of ciri-Full and circRNA-Full

Full-length circRNA sequences of the existing databases were constructed using a combination of all exons within the back-spliced junction sites. So far, there are no databases for the full-length sequences of experimentally validated circRNAs. There are two tools, isocirc and ciri-long, which both have a computational pipeline to reliably characterize full-length circRNA isoforms using long-read RNA sequencing data. The full-length circRNA sequences produced by isocirc and ciri-long were used as the validated full-length circRNA resources to compare our developed method, circRNA-full, with ciri-full.

We reconstructed full-length sequences using ciri-full and our method, circRNA-full. We used isocirc for human data and ciri-long for mouse data to obtain validated full-length circRNA resources, as the length of the long reads for human data was greater than 1000 bp and for mouse data the length of the long reads was less than 1000 bp. We determined the sequence to be correctly reconstructed if the sequence identified by ciri-full or circRNA-full was identical with the sequences identified by isocirc or ciri-long.

We used three accuracy measurements: precision, sensitivity and F1 score to compare the performance of ciri-full and circRNA-full. The precisions of circRNA-full (56.93, 57.69, and 58.93%) were higher than that of ciri-full (40.85, 40.52, and 41.28%) for the three samples in human data (Table 2). Again, the precisions of circRNA-full (22.14% and 21.31%) were higher than that of ciri-full (21.34% and 20.79%) for the two samples in mouse data. CircRNA-full achieved higher sensitivity (95.15, 94.89, and 94.95%) than ciri-full (79.35, 74.66, and 76.60%) for the three samples in human data. In the mouse data, circRNA-full also achieved higher sensitivity (99.67 and 99.78%) than ciri-full (97.47and 81.06%). The F1 scores of circRNA-full (0.7124, 0.7175, and 0.7272) were higher than that of ciri-full (0.5393, 0.5253, and 0.5365) for three samples in human data. Again, circRNA-full gained higher F1 scores (0.3623 and 0.3512) than ciri-full (0.3501 and 0.3309) for two samples in mouse data.

**Figure 4:**
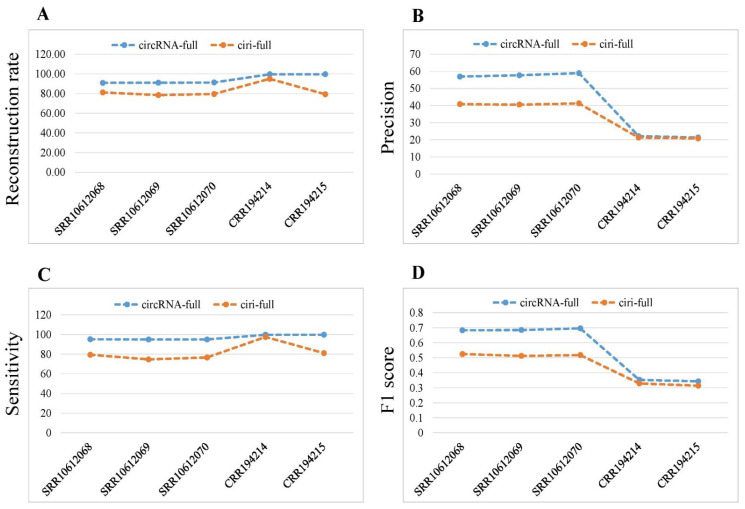
Performance comparison of circRNA-full and ciri-full. (**A**) Reconstruction rate. (**B**) Precision. (**C**) Sensitivity. (**D**) F1 score for different samples.

The reconstruction rate of circRNA-full was greater than that of ciri-full for human and mouse data (Figure 4A). The precision of circRNA-full was higher than that of ciri-full for all samples (Figure 4B). CircRNA-full achieved greater sensitivity than ciri-full for both the data sets (Figure 4C). CircRNA-full gained larger F1 score than that of ciri-full for all samples (Figure 4D). Overall, we found that our developed method, circRNA-full, showed better performance than ciri-full.

## 3. Discussion

There are several circRNA prediction tools, but a limited number of methods to reconstruct full-length circRNA sequences. Full-length circRNA sequence reconstruction is crucial to explore the functions of circRNA. We developed a new method, circRNA-full, for the reconstruction of full-length circRNA sequences utilizing the chimeric alignment information from STAR aligner. We validated our method with the reliable full-length circRNAs generated by isocirc and ciri-long from the long reads RNA sequencing data.

We reconstructed full-length sequences by our method, circRNA-full, and using ciri-full for human and mouse data. We found that circRNA-full reconstructed more full-length circRNA sequences (in percentage) compared to ciri-full. More full-length circRANs were produced in mouse data than human as the read lengths of mouse data were longer than human. CircRNA-full produced longer full-length circRNA sequences compared to ciri-full in both human and mouse data. The number of back-spliced reads was greater in mouse data than in human data for the full-length circRNA reconstructed by both circRNA-full and ciri-full.

For full-length circRNA sequence reconstruction, a limited number of approaches are available, including ciri-full, FUCHS, and FcircSEC. Ciri-full uses both BSJ (back-splice junction) sites and RO (reverse overlap) properties and require sequencing data to assemble full-length circRNA sequences. The main drawback of ciri-full is that it is not applicable if the sequencing read lengths for all reads in the RNA-seq data are not equal. FUCHS characterizes putative full-length circRNA sequences using RNA-seq information from long reads (>150 bp). It is not suitable for short reads and doesn’t provide the full-length sequence directly. FcircSEC is designed for full-length circRNA sequence reconstruction and classification using the annotation information of the reference genome. It depends fully on annotation information, and sequence information is not utilized in this method. Our developed method circRNA-full is free from the limitations mentioned in the existing methods.

We compared the performance of circRNA-full and ciri-full based on precision, sensitivity and F1 score. The reconstruction rate of circRNA-full was higher than ciri-full in both human and mouse data. CircRNA-full achieved higher precision, sensitivity and F1 score than ciri-full in both human and mouse data. Thus, we can conclude that our developed method, circRNA-full, performed better than ciri-full.

We evaluated our method by comparing the sequences produced by our method with the sequences produced by isocirc/ciri-long. We assume that the sequences produced by isocirc and ciri-long are correct. The methods isocirc and ciri-long utilize long reads RNA-seq data; these data are relatively low throughput and the error rate of long read sequencing technology is relatively high. Therefore, isocirc and ciri-long may produce incorrect sequences sometimes, which may affect slightly the evaluation metrics of our method. This issue does not affect the performances of our method much because the evaluation metrics of our method are relatively high compared to the existing method, ciri-full.

## 4. Materials and Methods

Our method, circRNA-full, requires five types of input: (1) the output of the circRNA prediction tool CIRCexplorer, (2) chimeric alignments (in bam) produced by STAR, (3) chimeric junction (junction file) generated by STAR, (4) a reference genome, and (5) annotation of the reference genome. For each circRNAs, we extracted the individual alignments of the spanning reads of the circRNA. From the individual alignments, we extracted exons and introns utilizing the CIGAR value of the alignments. Then, we detected skip exons (if any) defined as the intersection part between exons and introns. Finally, the skip exons were deleted from the exons set and the remaining exons composed the full-length sequence. We compared the performance of our method, circRNA-full, with the existing method ciri-full based on precision, sensitivity and F1 score. As there is no resources/databases for the experimentally validated circRNAs, we used two tools, isocirc [5] and ciri-long [36], to build comparatively reliable validated circRNA resources to compare our method. Both of these tools, isocirc and ciri-long, are computational pipelines to reliably characterize full-length circRNA isoforms using long-read RNA sequencing data. We defined a sequence reconstructed by ciri-full/circRNA-full as correct if it was identical with the sequences identified by isocirc or ciri-long.

### 4.1. Data Description

The short reads NGS (next generation sequencing) RNA-seq data were downloaded from the Sequence Reads Archive (accession number: SRR10612068, SRR10612069, SRR10612070) and the National Genomics Data Center (https://bigd.big.ac.cn/gsa, accessed on 3 November 2021) (accession number: CRR194214, CRR194215). Long reads third generation sequencing (Nanopore sequencing) data were downloaded from the Sequence Reads Archive (accession number: SRR10612050, SRR10612051, SRR10612052, SRR10612053, SRR10612054, and SRR10612055) and the National Genomics Data Center (accession number: CRR194190, CRR194191, CRR194194, and CRR194195). Both the short reads and long reads downloaded from the same database were derived from same experiment samples. All the sequencing data downloaded from Sequence Reads Archive were derived from the cultured HEK293 cells, and all the data downloaded from the National Genomics Data Center were derived from adult mice. The reference genomes of human (GRCh38/hg38) and mouse (GRCm39/mm39) were downloaded from UCSC.

To evaluate the performance of our method, we compared the sequences produced by our method with experimentally validated circRNA sequences. In the literature there are no databases/resources for experimentally validated circRNA sequences, but there are two tools, isocirc and ciri-long, which can reliably characterize full-length circRNA isoforms using long-read RNA sequencing data. The limitations of long reads third generation sequencing data are that the error rate of these data are relatively high, and they are relatively low throughput. Despite having these limitations, we used long reads third generation data to obtain comparatively reliable full-length circRNA sequences.

### 4.2. Extract Alignments of Spanning Reads for Individual circRNA

In this step, we extracted the alignments of spanning reads for each circRNA using the output of CIRCexplorer and the chimeric alignment bam file produced by STAR. The circRNAs were produced by some spanning reads and in this step we extracted the alignment results of these spanning reads for each individual circRNA.

**Figure 5:**
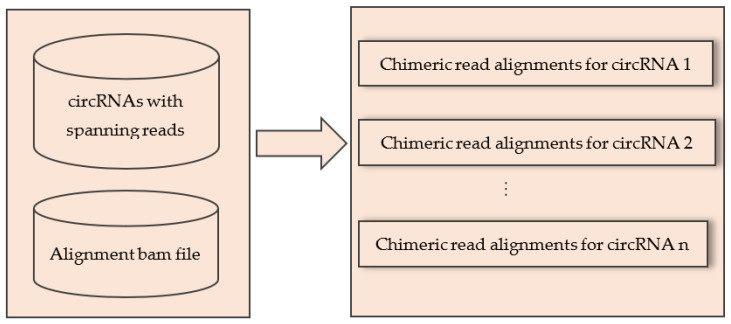
Extracting alignment of circRNA spanning reads for each circRNA from the alignment bam file.

The inputs of this step were: (1) the list of all circRNAs with the name of their spanning reads, and (2) the alignment bam file containing all chimeric reads. Input 1 was a two-column txt file where the first column contained chromosome, start and end position of circRNAs separated by “:” and “|”. The second column contained the list of spanning reads of the circRNAs separated by commas. Input 2 was the standard alignment bam file containing only the chimeric reads. The output of this step generated a separate txt file for each circRNA that contained the alignment of chimeric reads that spanned that circRNA. The pictorial representation of this step was given in Figure 5.

### 4.3. Extract Exons/Introns from the CIGAR Value of Bam File

In this step, we extracted exons from the CIGAR value of the chimeric read alignment bam file. We utilized the CIGAR string M and N to extract exons and introns, respectively, where M represented matches in exons and N represented skip elements due to introns. For example, if the alignment start position at POS=125314967 and CIGAR=67M8354N57M7713N81M44S, then the procedure of extracting exons/introns was as follows. Let us define,

$$s_{i}=\left\{\begin{array}{c} POS\;for\;i=1\\ \;e_{i-1}+1\;for\;i=2,3,\ldots \end{array}\right.\;e_{i}=s_{i}+n_{i}-1\;for\;i=1,2,\ldots$$

where $n_{i}$ is the number corresponding to CIGAR string, $s_{i}$ is the start position and $e_{i}$ is the end position of the corresponding exon or intron.

In Table 3, M represents the exons and columns 6 and 7 show the corresponding exon start and end positions. On the other hand, N represents introns and columns 3 and 4 show the corresponding intron start and end positions. The CIGAR value also contained the characters such as S, H, D, and I. However, we were not interested in these characters and they had no effect on our method. Extraction of exons was not fully dependent on CIGAR string. The exons obtained from CIGAR string were compared with exons in the gene annotation information. If any exon obtained from CIGAR string intersected with an exon in the reference genome annotation, the exon from annotation was selected as final exon.

**Figure 6:**
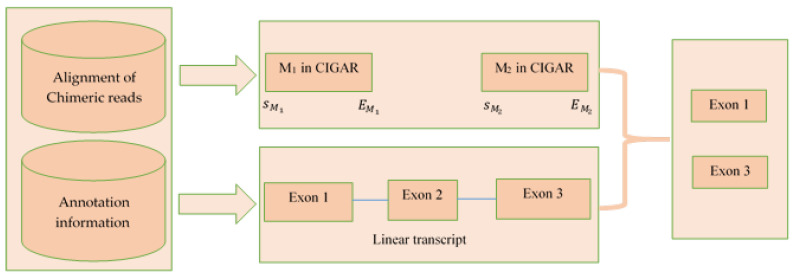
Procedure of extracting exons from the CIGAR string from the alignment of chimeric read.

The inputs of this step were: (1) alignments of chimeric reads for each circRNA and (2) the annotation file (corresponding to reference genome). The CIGAR value of the chimeric read was used to extract the exons. Input 1 was a 13 column txt file containing the alignment information (alignment start position, cigar value etc.). Input 2 contained the coordinate of each exon from the annotation file. Input 1 contained the alignment start position for each chimeric read, and the alignment end position was obtained using the CIGAR information. Then, from input 2, the exons having some intersection with the alignment start and end position of CIGAR string M of the chimeric read were selected as final exons set. Figure 6 is a pictorial representation of this step.

### 4.4. Detecting Skip Exons

**Figure 7:**
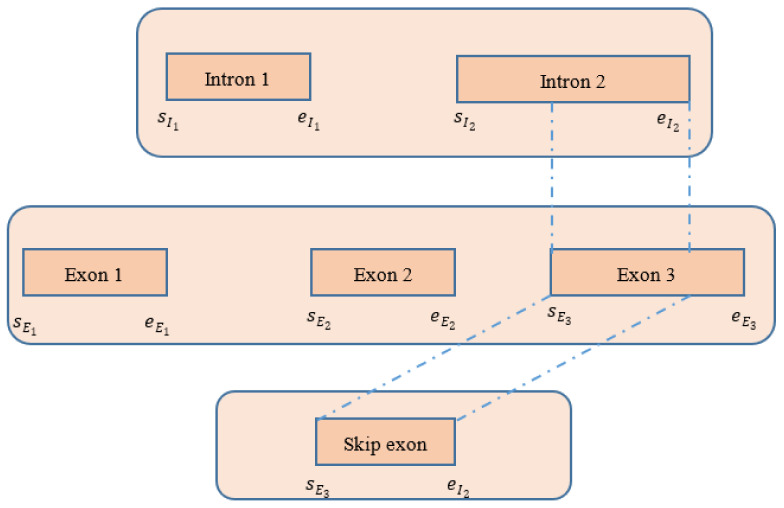
Procedure of detecting skip exon after extracting exons and introns from the CIGAR string of the spanning chimeric reads.

The procedure of extracting exons/introns from the CIGAR string is described in Table 3 in Section 4.3. After obtaining the introns, we investigated whether there was any intersection between the introns and the exons. If we found any intersection between exons and introns, we defined the intersection part as a skip exon. A full exon or a part of an exon might be detected as a skip exon. The detailed procedure is shown in Figure 7.

### 4.5. Deletion of Skip Exon and Reconstruction of Full Sequence

**Figure 8:**
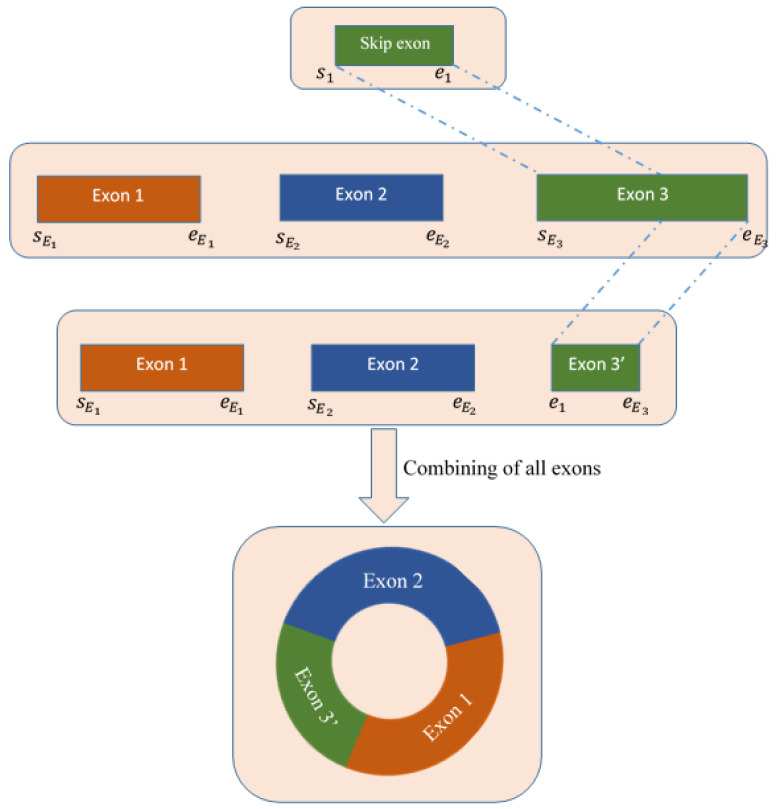
Procedure of deleting skip exon from all available exons extracted from the CIGAR string of the spanning chimeric reads.

We extracted the exons and introns utilizing the CIGAR value of the chimeric alignments of the spanning reads of the circRNA. Then, we detected the skip exon (if any) as the intersection part of exon and intron. In this step, we deleted the skip exon from the available exons and obtained the final exons set. Finally, combining these exons sets, we reconstructed the full-length sequence of circRNAs. The detailed procedure is shown in Figure 8.

### 4.6. Identification of circRNAs and Reconstruction of Full-Length Sequences

We used two popular circRNA prediction tools, CIRI (version 2) and CIRCexplorer (version 2), for the identification of circRNA. For mapping the sequencing reads to the genome, we used BWA [37] for CIRI, and STAR [38] for CIRCexplorer with default parameters. For the reconstruction of full-length circRNA sequences, we used ciri-full and our developed method, circRNA-full. Both of these two methods required information of identified circRNAs and sequencing reads as input.

### 4.7. Evaluation Criteria for Performance Comparison

We reconstructed full-length circRNA sequences using long-reads (produced by Nanopore technology) as validated circRNA sequences because no databases for experimentally validated circRNAs are available in the literature. We assessed the reconstructed sequences produced by short reads compared with the sequences produced by long reads, provided that both the short reads and long reads were derived from the same sample.

So far, in the literature, two tools isocirc and ciri-long, are available for reconstructing full-length circRNA sequences using long reads. These tools are used to reliably characterize full-length circRNA isoforms using long-read RNA sequencing data. A sequence reconstructed by short reads was considered correct if it was similar to any one of the sequences produced by isocirc. Similarly, a reconstructed sequence produced by short reads was declared correct if it was similar to any of the sequences produced by ciri-long. The similarity of the sequences produced by short reads and long reads was defined according to criteria 1 and 2.

criteria 1. *A* and *B* have more than 95% similarity
criteria 2. $\left|M-average\left\{l\left(A\right),\;l\left(B\right)\right\}\right|<10$
where *A* and *B* are the sequences produced by short reads and long reads, respectively, M is the number of matched elements between *A* and *B*, and $l\left(A\right)$ and $l\left(B\right)$ are the length of *A* and *B* respectively.

The reconstructed full-length circRNA sequences verified by long reads were defined as true positives, whereas those not verified by long reads were defined as false positives. The full-length circRNA sequences that were verified by long reads in other reconstruction method (e.g., ciri-full) but not assembled in current reconstruction method (e.g., circRNA-full) were defined as false negatives in the current reconstruction method. The evaluation metrics used for comparing the performance of circRNA-full with ciri-full are defined as follows:

$$Precision=\frac{TP}{TP+FP}$$

$$Sensitivity=\frac{TP}{TP+FN}$$

$$F1\;Score=\frac{2*precision*sensitivity}{precision+sensitivity}$$

where *TP*, *FP* and *FN* are the number of true positives, false positives and false negatives, respectively.

## 5. Conclusions

Several methods are available in the literature for the identification of circRNAs. However, a limited number of methods are available for the reconstruction of full-length circRNA sequences. We developed a new method, circRNA-full, for full-length circRNA sequence reconstruction incorporating chimeric alignment information. We evaluated the performance of our method with full-length circRNA sequences produced by isocirc and ciri-long using long-reads RNA-seq data. Our method, circRNA-full, had a better reconstruction rate, precision, sensitivity and F1 score than the existing full-length circRNA sequence reconstruction tool, ciri-full, for both human and mouse data. We developed an R package for our method which is freely available at https://github.com/tofazzalh/circRNAFull.

## Figures and Tables

**Table 1:** Number of reconstructed sequences produced by ciri-full and circRNA-full for different samples.

Species	Sample	NSC	ciri-Full		circRNA-Full	
			NRS	% of RS	NRS	% of RS
*Homo Sapiens*	SRR10612068	3756	3048	81.15	3411	90.81
	SRR10612069	3254	2552	78.43	2959	90.93
	SRR10612070	3290	2616	79.51	3002	91.25
*Mus Musculus*	CRR194214	9699	9209	94.95	9658	99.58
	CRR194215	10,911	8649	79.27	10,864	99.57

Note: NSC = Number of sequences used for comparison, NRS = Number of reconstructed sequences, RS = Reconstructed sequences.

**Table 2:** Performance comparison of ciri-full and circRNA-full using different accuracy measures.

Species	Sample	Method	NRS	TP	FN	Precision	Sensitivity	F1 Score
*Homo sapiens*	SRR10612068	ciri-full	3048	1245	324	40.85%	79.35%	0.5393
		circRNA-full	3411	1942	99	56.93%	95.15%	0.7124
	SRR10612069	ciri-full	2552	1034	351	40.52%	74.66%	0.5253
		circRNA-full	2959	1707	92	57.69%	94.89%	0.7175
	SRR10612070	ciri-full	2616	1080	330	41.28%	76.60%	0.5365
		circRNA-full	3002	1769	94	58.93%	94.95%	0.7272
*Mus Musculus*	CRR194214	ciri-full	9209	1965	51	21.34%	97.47%	0.3501
		circRNA-full	9658	2138	7	22.14%	99.67%	0.3623
	CRR194215	ciri-full	8649	1798	420	20.79%	81.06%	0.3309
		circRNA-full	10864	2315	5	21.31%	99.78%	0.3512

Note: NRS = Number of reconstructed sequences, TP = True positives, FN = False negatives.

**Table 3:** Process of extracting exons and introns from the CIGAR value of chimeric alignments.

CIGAR String	Corresponding Number $n_{i}$	Start $s_{i}=e_{i-1}+1$	End $e_{i}=s_{i}+n_{i}-1$	Transcript Name	Intersecting Exon Start	Intersecting Exon End
M	67	125314967	125315033	NM_021964	125313307	125313656
N	8354	125315034	125323387			
M	57	125323388	125323444	NM_021964	125323308	125323444
N	7713	125323445	125331157			
M	81	125331158	125331238	NM_021964	125331157	125331238

## Data Availability

Not applicable.
